# Effect of *Bacillus velezensis* to substitute in-feed antibiotics on the production, blood biochemistry and egg quality indices of laying hens

**DOI:** 10.1186/s12917-020-02570-6

**Published:** 2020-10-23

**Authors:** Miao Ye, Chunjie Wei, Anam Khalid, Qian Hu, Ru Yang, Binghong Dai, Hengwei Cheng, Zaigui Wang

**Affiliations:** 1 College of Life Science, Anhui Agricultural University, No. 130, Changjiang Road, Anhui 230036 Hefei, The People’s Republic of China; 2 Department of Animal Science, Purdue University, 270 S, Russell ST, IN 47907 West Lafayette, USA

**Keywords:** *Bacillus velezensis*, Production performance, Egg quality, Serum biochemical index

## Abstract

**Background:**

The excessive use of antibiotics in the livestock feed industry caused inevitable side effects of microbial resistance. Besides this residual antibiotics in animal-derived foodstuff imposed serious health problems for humans. So this study aimed to investigate the potential use of *Bacillus velezensis* to substitute antibiotics for poultry production. A total of 468, 49-week-old Hy-Line Brown chickens, were randomly divided into four groups the control group (regular diet), experiment group I (0.1% *B. veleznesis*), experiment group II (0.2% *B. veleznesis*), and antibiotic group (50 mg/kg flavomycin), with three replicates per group and trial period consisted on 42 days.

**Results:**

The results showed that, compared with the control group, the average egg production rate and daily feed intake of experimental groups I and II increased significantly (*P* < 0.05), while the average egg weight was increased in experimental group II as compared to (I) (*P *< 0.01). The feed conversion ratio was decreased (*P* > 0.05) in group (II) Egg quality parameters such as yolk weight of the experimental group II was increased, but that of the antibiotic group and experiment group I was decreased, neither significant (*P* > 0.05). Moreover, the eggshell strength, yolk color, albumen height, and Haugh unit were significantly increased (*P* < 0.05). Compared with the control group, probiotic groups can increase the progesterone and motilin (*P* > 0.05) but decrease the secretin and cholecystokinin in the blood plasma (*P* > 0.05).

**Conclusions:**

This study suggested that *B. velezensis* can substitute in-feed-antibiotics and improved most of the study parameters significantly. Which suggested *that B. velezensis* has potential future application value to replace the feed antibiotics.

## Introduction

Antibiotics are chemical substance produced by microorganisms which can resist pathogens to improve human and animal health as well as improve the quality of food products. Since the discovery of antibiotics in 1929, they have contributed to treat infectious diseases previously known to kill many humans and animals. Therefore, antibiotics are called guardians of human beings [1–3]. A class of antibiotics (Spiramycin, Tetracycline, Virginiamycin, Erythromycin) has been added to poultry feed for enhancing growth and production in large-scale intensive farming environments [4]. Adding a certain amount of feed antibiotics can not only promote the feed conversion rate, growth, development of poultry and reduce feed to egg ratio but also increases economic benefits, leading to expand the modern intensified poultry industry [5, 6].

However, with the continuous development of animal husbandry, the problem of antibiotics abuse has also emerged. Antibiotics remaining in livestock and poultry products (such as meat, eggs, and milk) may induce abnormal reactions such as disturbances in physiological and biochemical processes [7]. Some studies, for example, have shown that the abuse of antibiotics has a certain degree of correlation with the increased risk of cancer [8, 9]. The Use of antibiotics in animal production imposes a serious selection pressure on microbes which are exposed to sub-inhibitory doses of antibiotics. This has raised the problem of antibiotic resistance through mutation and gene transfer [10]. Controlling the propagation of antibiotic-resistant bacteria has become a major health problem in the 21st century [11]. Besides, a high proportion of antibiotics added to animal feed is excreted in animal’s urine or manure, causing water pollution which could be great harm to water sources and human health ultimately [12, 13]. Effective intervention are required, to reform the regulatory environment and to reclassify the antibiotics, by putting some antibiotics as a treatment choice and all others strictly restricted is the suggested solution for all above problems in the countries where antibiotics are available without a prescription[14]. Similar to the successful regulation of veterinary medicine in the Republic of Korea [15]. Also, antibiotics in animal feed have been completely banned by the Chinese government from July 1, 2020. However, it is feared that banning the use of antibiotics may have adverse effects on animal health and farmers’ profits. This has led efforts from all over the world to find an animal growth promoter that can effectively replace antibiotics to prevent antibiotic residues in poultry products, such as eggs [16, 17]. Currently, probiotic preparations, Oligosaccharide preparations, enzyme preparations, and Chinese herbal medicine are effective antibiotic substitutes [18–21]. Probiotic preparations have become an emerging growth-promoting additive due to the enormous amount of research and good effect on animal growth [22, 23].

*Bacillus velezensis* has been widely used as a biological control agent in agricultural fields due to its excellent ability to suppress plant diseases [24, 25]. It is considered as a potential rhizobacterial organism with extraordinary biosynthetic machinery, which can trigger innate immunity in plants [26].

However, there are few experiments on the *B. velezensis* used as feed antibiotics replacement [24]. It was suggested that this bacteria can inhibit adherence, replication, and virulence of intestinal pathogens [27]. In addition, it can play an important role to modulate the immune system [28]. So, we prognosis that *B. velezensis* have the potential to be developed as a probiotic agent. In the present experiment, to prove that the *B. velezensis* can be a good antibiotics substitute, we investigated the effect of adding 1.0 × 1010 CFU/kg and 2.0 × 1010 CFU/kg of *B. velezensis* as a feed additive instead of flavomycin on the production performance, egg quality, blood physiological and biochemical indices of Hy-Line Brown laying hens. To the best of our knowledge, this is the first time *B. velezensis* is assayed to replace antibiotics in hens feed. This study provided a practical basis for the application of *B. velezensis* to the laying hens feed instead of antibiotics.

## Results

### Production performance

As shown in Table 1, The average egg production rate in the experiment group I, and II were increased significantly as compared to the control group (*P* < 0.05), however, it decreased as compared to the antibiotic group. The average egg weight (AEW) was increased significantly in experimental group II as compared to the experimental group (I) The average daily feed intake of the antibiotic group, experimental group I, and experimental group II increased significantly (*P* < 0.01), (*P* < 0.01), (*P* = 0.045 < 0.05). There were no treatment effects on the feed conversion ratio of the control group, antibiotic group and the experimental group I, however, it reduced significantly in group (II) (*P* < 0.01).

**Table 1:** The effect of *Bacillus velezensis* on the production performance of laying hens

Treatment	Control group	Antibiotic group	Experiment group I	Experiment group II
AEPR (%)	78.889 ± 0.007^c^	84.596 ± 0.006^a^	80.827 ± 0.005^b^	81.905 ± 0.006^b^
AEW (g)	61.972 ± 0.150^a^	61.045 ± 0.277^b^	60.362 ± 0.140^c^	61.192 ± 0.111^b^
ADFI (g)	110.608 ± 0.368^d^	113.830 ± 0.257^a^	112.546 ± 0.281^b^	111.435 ± 0.229^c^
FCR	2.295 ± 0.022	2.241 ± 0.020	2.323 ± 0.017	2.257 ± 0.021^a^

*AEW* Average egg weight, *ADFI* Asverage daily feed intake, *FCR* Feed conversion ratio

^a^, ^b^, ^c^, ^d^ different lower case superscript showed significant difference in a row (*p *< 0.05)

### Egg quality

The egg quality was determined twice in the whole period. As presented in Table 2, in the first determination on day 21 of the phase one (2–21 days), compared with the control group and antibiotic group EW, ESI and ESW in both experimental groups was slightly decreased, but not significant (*P* > 0.05). However, a significant decrease in ESS was observed in both experimental groups as compared to the control and antibiotics group. And the eggshell color ESC, YW, YC, AH, and HU were higher than that of the control group but lower than that of the antibiotic group.

**Table 2:** The effect of *Bacillus velezensis* on the egg quality of laying hens

	Treatment	Control group	Antibiotic group	Experiment group I	Experiment group II
Egg quality index of the first phase	EW (g)	62.168 ± 0.801	63.154 ± 1.141	61.659 ± 0.537	58.968 ± 3.766
	ESI	1.312 ± 0.007	1.312 ± 0.005	1.299 ± 0.008	1.305 ± 0.008
	ESC	26.234 ± 0.491	27.752 ± 1.444	27.110 ± 0.005	26.511 ± 0.414
	ESS (× 10^5^Pa)	4.227 ± 0.086^a^	4.147 ± 0.019^a^	3.832 ± 0.117^b^	3.942 ± 0.103^ab^
	YW (g)	15.835 ± 0.293	16.205 ± 0.167	16.006 ± 0.165	15.901 ± 0.732
	ESW(g)	8.941 ± 0.138	8.803 ± 0.101	8.651 ± 0.155	8.675 ± 0.119
	YC	6.363 ± 1.028	7.713 ± 0.560	7.365 ± 0.771	7.302 ± 0.844
	AH (mm)	6.345 ± 0.673	7.733 ± 0.122	6.946 ± 0.828	6.962 ± 0.709
	HU	75.497 ± 5.646	86.621 ± 0.864	80.701 ± 6.826	82.039 ± 6.139
	TG (mg/g)	79.455 ± 8.725	102.570 ± 7.959	136.104 ± 4.681	124.865 ± 6.275
	CH (mg/g)	17.736 ± 0.312	16.713 ± 0.499	17.177 ± 0.307	17.059 ± 0.617
Egg quality index of the second phase	EW (g)	63.521 ± 0.290	61.328 ± 1.364	60.575 ± 0.298	61.726 ± 1.449
	ESI	1.316 ± 0.003	1.307 ± 0.006	1.314 ± 0.008	1.318 ± 0.013
	ESC	25.011 ± 0.593	23.507 ± 0.172	26.638 ± 1.958	24.901 ± 0.497
	ESS (× 10^5^Pa)	3.397 ± 0.066	3.868 ± 0.053	4.027 ± 0.173	3.776 ± 0.147
	YW (g)	16.011 ± 0.125	15.486 ± 0.342	15.714 ± 0.134	16.144 ± 0.322
	ESW (g)	9.096 ± 0.206	8.747 ± 0.270	8.724 ± 0.079	8.542 ± 0.247
	YC	6.834 ± 0.190^b^	7.203 ± 0.245^ab^	7.330 ± 0.113^ab^	7.403 ± 0.099^a^
	AH (mm)	6.933 ± 0.021^c^	8.356 ± 0.170^a^	8.116 ± 0.073^a^	7.521 ± 0.178^b^
	HU	80.464 ± 1.378^c^	90.467 ± 0.787^a^	89.454 ± 0.415^a^	85.036 ± 1.606^b^
	TG (mg/g)	287.995 ± 12.579	255.341 ± 44.683	278.300 ± 12.529	306.650 ± 53.577
	CH (mg/g)	25.751 ± 2.445	24.413 ± 2.700	25.317 ± 1.323	18.805 ± 1.457

*EW* Egg weight, *ESI* Egg shape index, *ESC* Eggshell color, *ESS* Eggshell strength, *YW* Yolk weight, *ESW* Eggshell weight, *YC* Yolk color, *AH* Albumen height, *HU* Haugh Unit, *TG* Triglyceride, *CH* Cholesterol

^a^, ^b^, ^c^ Different lower case superscript were significant different in a row (*p* < 0.05)

In the second determination on day 42 of phase two (22–42 days) compared with the control group and antibiotic group the EW, ESW, and ESI had no significant difference in both experimental groups. And compared with the control group, the ESS and YC of experimental groups were not improved significantly (*P* > 0.05). ESC of the experimental group I was increased, The YW of the experimental group II was increased, but that of the antibiotic group and experimental group I was decreased, neither significant (*P* > 0.05). Moreover, the ESS, AH, and HU of the experimental groups were higher than that of the control group but lower than that of the antibiotic group. As presented in Table 2, antibiotics and *B. velezensis* could increase triglyceride in the first phase. However, triglyceride was reduced in the antibiotic group and experimental group I but increased in experimental group II in the second phase. Compared to the antibiotic, feed with 0.2% *B. velezensis* could reduce the content of cholesterol in the second phase.

### The effect of *B. velezensis* on biochemical indices in blood plasma

As shown in Table 3, triglyceride in plasma of antibiotic group and experimental group I was increased, but reduced in experimental group II, however none was significant (*P* > 0.05). The level of progesterone and motilin in the blood plasma of laying hens was increased in experimental group I and II as compared to those of the control group and antibiotic group, which recommended the successful usage of *B. velezensis*. The results also showed that the, the level of secretin and cholecystokinin was decreased in all three test groups as compare to the control group. All the above increase and decrease in the results were not significant (*P* > 0.05).

**Table 3:** The effect of *Bacillus velezensis* on the hormonal level in blood plasma of laying hens

Treatment	Control group	Antibiotic group	Experiment group I	Experiment group II
TG (mmol/dL)	1.191 ± 0.118	1.231 ± 0.147	1.404 ± 0.392	1.164 ± 0.019
CH (mmol/dL)	0.342 ± 0.013	0.389 ± 0.020	0.341 ± 0.011	0.323 ± 0.026
PROG (pmol/L)	531.444 ± 59.618	458.032 ± 34.864	596.202 ± 29.885	734.448 ± 129.771
MTL (pg/mL)	378.54 ± 24.24	343.528 ± 10.975	380.24 ± 27.81	515.70 ± 75.49
SC (pg/mL)	23.517 ± 5.784	19.056 ± 1.005	19.129 ± 0.833	23.485 ± 3.135
CCK (pg/mL)	1139.644 ± 45.681	883.897 ± 50.343	999.062 ± 103.643	1072.317 ± 119.428

*TG* Triglyceride, *CH* Cholesterol, *PROG* Progesterone, *MTL* Motilin, *SC* Secretin, *CCK* Cholecystokinin

## Discussion

### Production performance

The use of bacillus-based probiotic feed-formulation was observed to be a promising health-promoting approach. Bacillus spp. were widely used in the poultry industry [29].

At the late stage of feeding, due to the change of metabolism in the body, the absorption of nutrients in the feed was weakened, which lead to the decrease in production performance and the deterioration of egg quality, even the decrease of immunity and the deterioration of anti-stress ability, thus affecting economic benefits. Bacillus sp. such as *B. coagulans* has the functions of regulating or maintaining intestinal micro-ecological balance, enhancing immunity, promoting the absorption of calcium, phosphorus, vitamin D, and so on [30]. Numerous studies have shown that adding Bacillus sp. to the laying hen’s feed can significantly increase egg production rate, average egg weight, reduce feed conversion ratio, and improve egg quality and immunity [31–34]. Because eggs are the main products in the laying hen farm, and the egg production rate determines the breeding efficiency. Therefore, improvement of the egg production has an important economic value [35]. In this study, compared with the control group, the average egg production rate in the antibiotic group, experimental group I, and experimental group II were increased significantly (*P* < 0.01). Similar results reported that laying hens fed with *B. subtilis* OFMCC 1.921 had an improved egg production between week 5 to 8 and weeks 9 to 12 [34]. Another study suggested the similar result [36]. In addition, the results from that study also proved that *B. amyloliquefaciens* B-1895 improved the average egg production rate. However, Li et al. (2006) reported that hens fed with 3 × 109 CFU/g, 6 × 109 CFU/g, and 9 × 109 CFU/g of *B. subtilis* had no significant effect on egg production rate, but could significantly reduce average daily feed intake and feed conversion ratio with an improved production performance. However, it might be related to the Bacillus sp., treatment level, trial duration, variety, and age of laying hens [32].

As for the ADFI of the antibiotic group, the experimental group I, and experimental group II increased significantly (*P *< 0.05). And the feed conversion ratio of experimental group II was reduced significantly as compared to the control group (*P* < 0.01). However, Ribeiro et al. (2014) showed that feeding with *B. subitils* can increase the egg production rate by 2.63%, but there was no significant change in the feed conversion ratio [37]. In our study, the supplementation of *B. velezensis* could increase the average daily feed intake of experimental group I and reduce the feed conversion ratio of experimental group II. This may be due to the fact that probiotics can consume excess oxygen in the intestine, to produce bacteriocins and volatile bacteriostatic substances [38]. Which is conducive to the degradation of nutrients in feed and the improvement of feed conversion rate, thereby improving animal feed intake and reducing feed conversion ratio.

### Egg quality

The egg quality was determined twice in the whole period. The strength and thickness of eggshell are two important indicators to measure the quality of eggs. Because, the strength and thickness of eggshell also affect the freshness of eggs. Improving eggshell quality is important to the laying hen production, and it is also a research hotspot for domestic and foreign researchers at present. Many studies suggested that adding Bacillus sp. to the diet of hens can increase the eggshell thickness and eggshell strength, improve the quality of eggshell, reduce the rate of broken eggs, and improve the economic benefits of poultry farms [35, 39]. Similarly, it was also reported that the addition of *B. subtilus* to the diet improved the haugh unit and protein index significantly, and the thickness of eggshell was also improved [36]. Forte et al. (2016) proved that the using of 0.05% *B. subtilis* culture in the diet had the greatest effect on the egg’s physical characteristics and the yolk weight, shell weight, haugh unit, and color all increased significantly (*P* < 0.05) [40]. Similar results were obtained by [41]. However, in our study, at the second determination level, except for the eggshell strength, yolk color, albumen height, and haugh unit, there was no significant change in other indicators. It may be related to the late laying stage of hens. The mechanism of improving egg quality by *B. velezensis* still needs further study.

From Table 2, it can be noted that the supplementation of *B. velezensis* can increase triglyceride in the yolk of the first phase. Triglycerides were increased in all three test groups but the maximum increase was observed in experimental group II. While the content of cholesterol in yolk was decreased in all three test groups as compared to the control group. Park et al. pointed out, that the fermented buckwheat as a feed additive could reduce the yolk triglyceride [42]. It was also reported that there was a decrease in yolk cholesterol in hens fed with the *B. subtilis* supplemented diet [32]. In the second determination of egg quality, the results of some indices were not identical with that of the first determination, it might be related to the age of laying hens, as reported by Travel et al. (2011) that the low quality eggs can arise in young birds, due to early ovulation [43].

### The effect of *Bacillus velezensis* on biochemical indices in plasma

Results showed that triglyceride in the plasma of the antibiotic group and experiment group I was increased, but decreased in the experimental group II (*P *> 0.05). In the study of Choi et al. (2018) supplementation of fermented brown seaweed in the feed could significantly increase triglyceride and cholesterol in the blood of laying hens (*P* < 0.05) [44]. But Zhao et al. (2013) reported that fed with fermented Ginkgo-leaves has an increased effect on triglyceride and cholesterol in blood plasma [45]. The triglyceride and cholesterol in animal serum are important to animal cells. Most tissues in the body can use triglyceride decomposition products to provide energy for metabolism [46]. Cholesterol is a precursor of many important hormones and vitamin synthesis. It is also a component of the animal brain, liver, and other important cells [47]. Studies have confirmed that high levels of blood plasma cholesterol in animals could increase the risk of atherosclerosis. Therefore, the intake of animal meat products with low triglyceride and cholesterol content is beneficial to human health [48]. In our study, fed with 0.2% *B. velezensis* could decrease the content of triglyceride and cholesterol in blood plasma. This is consistent with the results of [49, 50], which could be helpful for improving the quality of eggs.

The study found that the dietary supplementation of 0.1% *B. velezensis* and 0.2% *B. velezensis* increased the progesterone and motilin in the blood plasma of laying hens compared to those of the control group, but not significant (*P* > 0.05). However, progesterone and motilin level in the antibiotic group were decreased (*P* > 0.05) (Table 3), which indicated the usage of *B. velezensis* in the feed was prevalent. It is known that progesterone is an important steroid hormone and its main target organ is the uterus. In addition, it also acts on other tissues, including the brain, pituitary, breasts, and ovary [51]. Its function is to promote the growth, development, and differentiation of these organs, and participate in their functional regulation. Moreover, progesterone is also involved in regulating and transforming the proliferation and differentiation of some abnormal cells, such as breast cancer cells and ovarian cancer cells [52, 53]. Kim et al. proved that adding *B. subtilis* to the heifers feed could obtain similar results as presented in our study. In addition, the results also confirmed that progesterone, induced the level of cholesterol, and progesterone is relative to the feed efficiency [54], which is consistent with our experimental results. But the mechanism of action is not fully investigated. Motilin is a gut peptide, produced in the upper intestinal mucosa that induces strong contraction in the small intestine which can prolong the time of gastric emptying [55]. Tack et al. (2016) have confirmed that motilin-induced gastric phase III contraction may be a starvation signal during the digestive interval, which explains the cause of hunger in humans to some extent. Lack of motilin and gastric phase III contraction may be associated with unexplained loss of appetite [56]. According to the results of this experiment, we can conclude that supplementation with *B. velezensis* could increase the average daily feed intake, which was correlated with the increase of motilin.

In addition, *B. velezensis* in the feed can decrease the secretin and the cholecystokinin (*P *> 0.05). Secretin is the earliest discovered animal hormone, mainly distributed in the duodenal mucosa, a small amount in the jejunum, ileum, and antrum [57]. Since it was further reported that secretin is a kind of enterogastrone, it has attracted more and more attention [58, 59]. However studies confirmed that secretion has a strong inhibitory effect on gastric acid secretion in humans, dogs, and rats [60, 61], and hence should be reduced. In this experiment, adding *B. velezensis* to the laying hens feed could decrease secretin content and increase average daily intake, we inferred that secretin could inhibit gastric motility, if secreted abnormally in the plasma of the laying hens. Cholecystokinin was discovered in 1928 and it can induce gallbladder contraction and promote pancreatic enzyme secretion. Cholecystokinin acts as a satiety neurotransmitter to regulate the termination of feeding [62]. Under modern production conditions, inadequate feeding of animals is a common phenomenon. In order to develop the production potential of animals and to further improve the performance of animals, the first consideration is how to increase feed intake. Due to the important role of secretin and cholecystokinin in feeding regulation, reducing the content of secretin and cholecystokinin in animals has become a worth considering way to improve the feed intake.

It should be emphasized that although the alteration of the hormone in the plasma was not significant comparing to the control group. But, these hormones could play a great amplified role on the body, and also have a positive effects on the productivity of hens and the quality of eggs.

## Conclusions

The study concluded that dietary supplementation of with *B. velezensis* could improve the production performance, egg quality and the plasma biochemical index in poultry. As this was the first study to use *B. velezensis* as a replacement of antibiotics so, the results of experimental groups were not better than those of the antibiotic group, but to a certain extent, *B. velezensis* can replace the feed antibiotics, which has potential future applications to be used in animal feed industry.

## Methods

### Animals and grouping

A total of 468, 49-week-old healthy, Hy-Line Brown laying hens with similar weight, were received from the Institute of Animal Husbandry and Veterinary Medicine of Anhui Province. They were randomly assigned to 1 of 4 groups (control group, experiment group I, experiment II, and antibiotic group), three replicates per group with 39 hens per replicate. A Completely randomized design was used [63] to avoid any biasness of selection while allocating hens to the experimental groups.

Hens of the control group were fed a regular diet (59% yellow corn, 27.5% bean pulp, 8.5% limestone powder, and 5% premix, with 16% Crude protein, and 2556.0 Kcal/Kg Metabolic energy) without any active probiotic [32]. According to the results from our preliminary studies (unpublished data) [64], a new strain of *B. velezensis* was isolated from the manure of piglets. Hens of experimental group I and II were fed the regular diet plus 1 × 1010 CFU/kg and 2 × 1010 CFU/kg *B. velezensis*, respectively; and hens of the antibiotic group were fed the regular diet plus 50 mg/kg flavomycin.

### Feeding management

The laying hens were kept in a 3-tier bird’s cage (28 × 48 cm x 48 cm). To avoid contamination by pathogens aviary system was used [65], in which the cages were separately housed based on the treatment, under the same conditions, including room temperature, humidity, light, and ventilation, there was no enrichment provided for any environmental condition. Similar weight hens were used, and their health condition, feeding, and drinking were recorded daily, throughout the trial period and this strategy was used to reduce the effect of confounding variables. The laying hens were fed quantitatively twice a day [32], (7 am and 2 pm) for 42 days. This study was conducted at the Dongshan Chicken Farm of Anhui Academy of Agricultural Sciences; and the experimental protocol was approved by the Animal Care and Use Committee, the Anhui Academy of Agricultural Sciences, and the Ethics Committee of Anhui Agricultural University, Anhui, China.

### Data acquisition and analysis

Eggs were collected once a day (2 pm). The data for egg production, egg weight, and feed consumption were recorded. The trial period was divided into two phases (Phase 1: day 2–21 & Phase 2: day 22–42) to examine the production parameters based on the duration of feeding. Then, the average egg production rate (AEPR), average egg weight (AEW), average daily feed intake (ADFI), and feed/egg ratio (FCR) were calculated. On day 21 of phase 1 (day 2–21) and day 42 in phase 2 (day 22–42), 39 eggs from each treatment (balanced with egg weight) were collected and the egg quality indices including egg shape index (ESI), eggshell color (ESC), eggshell strength (ESS), yolk weight (YW), yolk color (YC), eggshell weight (EW), albumen height (AH), and Haugh Unit (HU) were determined. ESI was measured with a Vernier caliper (Measuring & Cutting Tool Work Co. China). ESC was measured with the eggshell color tester (Robotmation Com., Japan). ESS was tested with the Egg Shell Force Gauge MODEL-III (EGG-0530, Robotmation Com., Japan) [66]. Yolk weight, yolk color, eggshell weight, albumen height, and Haugh Unit were determined by using the Egg Multi Tester (EMT-5200 Robotmation Com., Japan). The egg yolk was collected in an aseptic centrifuge tube and stored at -20 °C.

On the last day of the experiment, a 2.0 mL blood sample from each randomly selected hen (1hen per cage, *n* = 39) was collected into a micro-anticoagulant tube [65]. The blood sample was centrifuged at 3000 rpm for 20 min at 4 °C by high-speed freezing centrifuge to obtain the plasma then stored at -20 °C until further analysis.

Blood concentrations of cholesterol and triglyceride were determined using cholesterol and triglyceride assay kits according to the manufacturer’s instructions (Changchun Huili Biotech, China). The progesterone, motilin, secretin, and cholecystokinin of egg yolk were determined using progesterone, motilin, secretin, and cholecystokinin kits, respectively, following the manufacturer’s instructions (Shanghai Enzyme-Union Biotech, China).

Since the study conducted, was based on a feeding trial and birds were not harmed during the whole trial period. After carefully collecting the blood sample, all the birds were healthy and returned to the cage again. No animals were killed at humane endpoints.

All the data were analyzed by using one-way analysis of variance (ANOVA) to determine whether any significant difference occurred within study groups or not. The data were presented as mean ± S.E.M, and the significance level was set up at *p* < 0.05 significant. The SPSS software, version 22.0 was used to analyze the data, and determine the effect of dietary treatments.

## Data Availability

Data used in this study will be available from corresponding author upon reasonable demand.

## Abbreviations

AEPR: Average egg production rate
AEW: Average egg weight
ADFI: Average daily feed intake
FCR: Feed conversion ratio
EW: Egg weight
ESI: Egg shape index
ESC: Eggshell color
ESS: Eggshell strength
YW: Yolk weight
ESW: Eggshell weight
YC: Yolk color
AH: Albumen height
HU: Haugh Unit
TG: Triglyceride
CH: Cholesterol
TG: Triglyceride
CH: Cholesterol
PROG: Progesterone
MTL: Motilin
SC: Secretin
CCK: Cholecystokinin
